# Storytelling training to promote stakeholder engagement in research dissemination

**DOI:** 10.1017/cts.2021.830

**Published:** 2021-08-09

**Authors:** Hae-Ra Han, Samuel Byiringiro, Cyd Lacanieta, Christine Weston, Mia Terkowitz, Melanie Reese, Michael Rosen, Cheryl Dennison Himmelfarb, Payam Sheikhattari, Michelle Medeiros, David Fakunle

**Affiliations:** 1 The Johns Hopkins School of Nursing, Baltimore, Maryland; 2 The Johns Hopkins Bloomberg School of Public Health, Baltimore, Maryland; 3 The Johns Hopkins Institute for Clinical and Translational Research, Baltimore, Maryland; 4 The Johns Hopkins School of Medicine, Baltimore, Maryland; 5 Older Women Embracing Life, Baltimore, Maryland; 6 Morgan State University, Baltimore, Maryland; 7 University of Maryland School of Medicine, Baltimore, Maryland; 8 DiscoverME/RecoverME: Enrichment Through the African Oral Tradition, Baltimore, Maryland

**Keywords:** Storytelling, dissemination, stakeholder engagement, research, training

## Abstract

Storytelling is increasingly recognized as a culturally relevant, human-centered strategy and has been linked to improvements in health knowledge, behavior, and outcomes. The Community Engagement Program of the Johns Hopkins Institute for Clinical and Translational Research designed and implemented a storytelling training program as a potentially versatile approach to promote stakeholder engagement. Data collected from multiple sources, including participant ratings, responses to open-ended questions, and field notes, consistently pointed to high-level satisfaction and acceptability of the program. As a next step, the storytelling training process and its impact need to be further investigated.

## Storytelling to Promote Stakeholder Engagement

Stakeholder engagement is defined as the process of meaningfully involving persons affected by research findings or programs in the research process.^1^ Recognized as a key process to improve the way research is prioritized, translated, and used in real-life settings,^2^ stakeholder engagement can occur across all stages of research, spanning from topic identification and study design to interpretation and dissemination of findings.^3^ A national survey^4^ revealed “research results disseminated to the community in a culturally relevant and appropriate manner” as one of the top indicators of successful community engagement.

The National Institutes of Health Clinical and Translational Science Award (CTSA) program emphasizes the importance of engaging nonacademic stakeholders in the research process. Therefore, many CTSAs have developed methods and infrastructure to support stakeholder engaged research.^5–7^ The Community Engagement Program of the Johns Hopkins Institute for Clinical and Translational Research (ICTR, Hopkins CTSA), which includes members representing three academic institutions and nonacademic stakeholders, collectively developed a training program using storytelling as a strategy for dissemination of research information and findings.

Storytelling or narrative communication (referred to as storytelling hereafter) is increasingly recognized as an effective, human-centered technique used among various settings and populations, such as older adults to improve cognition,^8,9^ patients with cancer, diabetes, and hypertension to improve their health behaviors,^10^ healthcare workers to address psychosocial stress,^11^ and community-level stigma reduction and awareness related to suicide.^12^ Recent systematic reviews highlight the positive impact of storytelling on health behaviors and outcomes among traditionally undervalued demographic groups, such as American Indian and Alaskan Native youth^13^ and African American women.^14^ Additionally, some researchers used storytelling as a stakeholder engagement approach to identify research priorities impacting young African American breast cancer survivors.^15^

Storytelling has a typical structure that includes the following:^16^ (1) cause-and-effect relationships between events that take place (2) over a certain period (3) that impact certain character(s). Different from traditional scientific communication aimed at providing abstract truths that are valid and context-free, storytelling is context-dependent as it attempts to derive a depiction and meaning of individual experiences from the cause-and-effect structure of the events. Within a sociocultural framework, storytelling is considered as a central medium of knowledge sharing and communicating science across diverse audiences in ways that make sense to the communities where the research takes place.^17,18^

## Educational Gap

Storytelling is considered highly relevant to populations with health disparities such as African Americans, for whom there is a strong cultural connection as a form of social discourse.^14^ While literature is growing to discuss “what” happened as a result of storytelling,^8–14,19^ published evaluations of storytelling training (i.e., how it was done and how it was received) are rare. Given the increasing attention to storytelling as a potentially versatile stakeholder engagement approach, it is important to describe the ways in which storytelling training is provided so that it can be adopted by future research teams as a practical engagement method. Therefore, this paper’s purpose is to discuss the format, scope, and evaluation outcomes of the storytelling training program offered by the ICTR’s Community Engagement Program.

## Target Audience

The target audience of the storytelling training program were faculty, research staff, community members, students, and fellows interested in utilizing storytelling approaches for disseminating research findings. The introductory seminar was advertised on Eventbrite, ICTR’s social media handles, through ICTR’s Community Engagement Program partners at the University of Maryland and Morgan State University, Hopkins’ School of Nursing network, emailing lists of existing community partner organizations, and to Hopkins’ Community Research Advisory Council members. The “how to” phase was a workshop designed to provide basic skills for creating individual stories and was primarily advertised to participants of the introductory storytelling seminar.

## Description of the Storytelling Training Program

Table 1 details the format, structure, and scope of the two-session storytelling training program which was offered over Zoom, an online video conferencing platform. The introductory seminar (first session) and skills building workshop (second session) were both facilitated by the same expert (DF), who has over 20 years of experience in the art of Black storytelling, African drumming, singing, and theater. Serving as a faculty and professor at two universities in the USA, the expert integrates the artistic and cultural practice to strengthen health, social equity, and liberation. Both sessions were designed to fit the needs of scholarly and community members wishing to share stories about what matters to them.

**Table 1.** Curriculum of storytelling training program

Phase 1: Introductory seminar		
Session		Length of time (min)
Welcome		5
Why storytelling		20
Core elements of storytelling		20
Talking research in real life		10
Questions and answers		5
Total		60

Phase 2: Storytelling skills building workshop		
Session		Length of time (min)
General introduction		15
Hero’s journey	Introduction	20
	Breakout session	20
	Debrief	15
Movie trailer	Introduction	10
	Breakout session	20
	Debrief	10
Story and the telling		10
Total		120

The purpose of the 1-hour introductory seminar was to orient participants to what storytelling is and how it can empower stakeholders. The presentation discussed how to portray one’s own personal context in a story, why storytelling matters, making the audience find themselves in a story, and the general elements that make a story a “story.”

The objective of the second session was to equip participants with the specific storytelling skills needed to better collaborate as researchers and community members, thus promoting community engagement in research. Not all participants had attended the introductory seminar, but the second session presented and dived deep into many of the concepts introduced in the first seminar. The second session began with the facilitator and each participant introducing themselves and expressing anything they enjoyed doing and/or were thankful for. Following introductions, the workshop consisted of two main activities: The Hero’s Journey and the Movie Trailer. The Hero’s Journey was used as the most common and popular template of storytelling that our participants can easily relate to.^20^ Through the Hero’s Journey, an individual’s evolving life story can be created to provide the person with meaning and purpose.^21^ The Hero’s Journey activity involved participants identifying where they would place themselves on a narrative progression map, which ranges from limited awareness of a problem to the mastery of a problem.^22^ This activity provided participants with a method for reflection on and framing of stories for prospective audiences. The movie trailer activity involved participants developing a short story analogous to a movie advertisement, applying the techniques introduced in the skills building workshop. The two main activities were structured as general introductions by the storytelling expert, followed by small group breakout sessions for hands-on practice and a full group debrief to discuss the experience. There were no specific story topics other than what interested the participants, and they were willing to share with their peers in the breakout rooms, and occasionally with the bigger group during debrief. Finally, participants convened to receive further elucidation on the “story” and “telling” components of storytelling and concluded with a question-and-answer period to address outstanding and critical points.

## Evaluation Methods

At the end of each of the two sessions, we administered a short survey to evaluate participants’ experience. The survey included multiple-choice, 5-point Likert-type scale, and open-ended questions. Given that this was an early and innovative program, the evaluation goals were largely formative and sought to characterize participants’ reactions and content of the process of the program. Additionally, qualitative evaluation of the skills building workshop utilized field notes captured during breakout sessions. Four notetakers were staff with the ICTR Community Collaboration Core and one was from University of Maryland Baltimore—a partner University for Community Engagement. Notetakers were each assigned to a breakout group solely to observe and document group interactions. Prior to the skills building workshop, notetakers were informed about the main objectives and briefed on good practices of field (albeit virtual) observations. Notetakers were instructed to document factual data (e.g., date, time, how many participants) along with participants’ interactions and conversational points.

Descriptive analysis was used to examine participants’ characteristics, as well as their ratings to the evaluation surveys administered after the introductory seminar and skills building workshop. Content analysis was used to summarize the main themes identified from the open-ended survey questions, and field notes pooled from the skills building workshop breakout groups.

## Results

### Workshop Session 1: Introduction to Storytelling

The introductory seminar received 289 registrants, and 110 attended the event on November 5^th^, 2020 (38% attendance rate). Fifty-six participants completed the evaluation survey (response rate = 51%), and feedback was mostly positive. When asked if they grasped the core elements of storytelling, 66% strongly agreed and 29% somewhat agreed. When asked if they appreciated the introductory seminar overall, 86% strongly agreed and 11% somewhat agreed. The qualitative feedback was also positive, with some respondents noting that the session was an “eye opener.”

### Workshop Session 2: Storytelling Skills Building

**Fig. 1.**
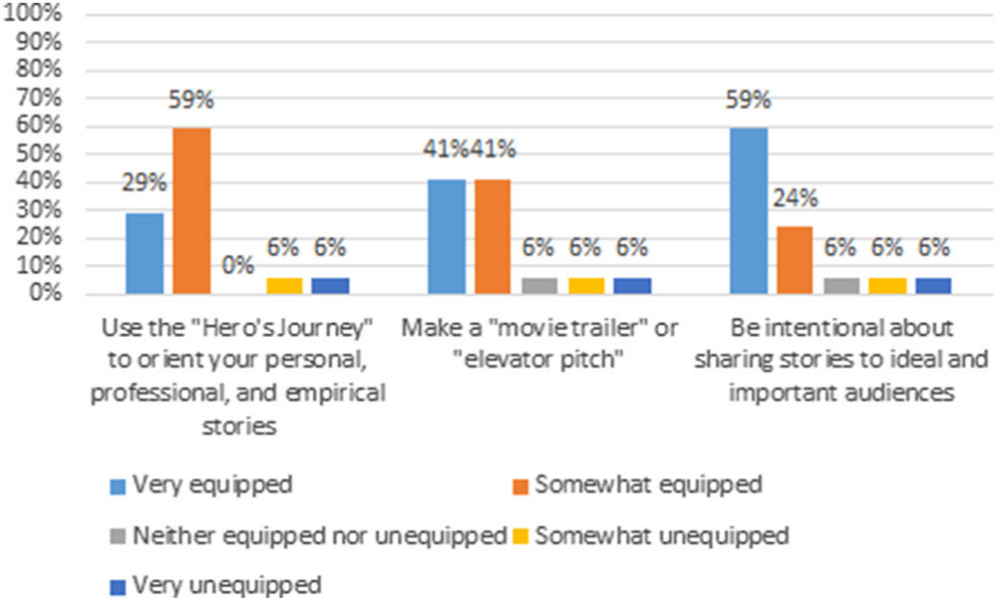
Storytelling participants’ responses to how they felt equipped to use “Hero’s journey,” “Make movie trailer,” and “be intentional about sharing stories to ideal and important audiences.”

The skills building workshop registration was capped at 50 individuals yet resulted in 60 registrants and 21 people attended (35% attendance rate). The workshop was held in two identically structured sessions on November 12 and 19, 2020. Participants were diverse, comprising of students and research fellows (52%), faculty and staff (38%), and community members (10%). Seventeen participants completed the evaluation survey (response rate = 81%). Among survey respondents, at least 80% felt very or somewhat equipped to use the “hero’s journey” to orient their personal, professional, and empirical stories, to make a movie trailer or elevator pitch, and to be intentional about sharing stories to relevant audiences (Fig. 1). Regarding the application of the skills learned from the workshop, 100% replied “strongly agree” or “somewhat agree” on committing to use the learned skills at their work, and they believed people they know could benefit from such workshop. Finally, all participants replied “strongly agree” that they appreciated the workshop overall.

Workshop participants’ written responses to open-ended questions about their experiences were notably positive, expressing that they learned tools to better communicate and help others to understand their work. Several participants wrote about their plans for using storytelling as a tool to engage stakeholders and as a method for disseminating their research to target communities and noted that it was a paradigm shift. Participants also appreciated the opportunity to interact with other participants while practicing their learned storytelling skills. One participant wrote:

*“This was the best workshop on Zoom that I’ve attended ALL YEAR. Thank you for keeping it so engaging and relevant. I think people often do not take the extra time and create the space needed to think about how to share their research in a way that is compelling/relevant/moving. Everyone in public health should take this workshop…”*

Field notes taken by trained staff included similar, generally positive responses shared verbally or observed in their breakout groups. Notetakers unanimously wrote that participants were highly engaged and showed passion for goals and topics. Participants were respectful of one another and were interested in each other’s stories. During the second breakout, some groups struggled with creating trailers but moved forward once their questions were answered by the instructor. One of the notetakers observed that the group seemed to have more energy when the instructor joined, and their discussion became more fluent.

## Lessons Learned and Moving Forward

Leveraging the growing emphasis and relevance of stakeholder engagement, the ICTR’s Community Engagement Program successfully developed and implemented a storytelling training workshop targeting researchers, research staff, community members, and students. According to evaluation data, using a two-phase approach (i.e., introduction to storytelling followed by a skills building workshop with interactive breakout activities) worked well for giving participants a general orientation about storytelling and followed by the opportunity to practice the art of storytelling. These findings are consistent with Urstad et al.^23^ who reported that storytelling generated strong emotional engagement while maintaining high levels of attention and interest from students in higher education. We observed that the learner-to-learner interaction increased communication and collaboration among participants. Such educational experiences have been shown to facilitate quality learning experience with significant positive outcomes for students.^24^

Despite the positive impact of the training program, some of the participants noted uncertainty about what they were expected to do or how to frame the stories. For future iterations, we will need to consider spending more time to review the process of developing stories with clearer instructions and modeling to foster a better understanding of the activities’ purposes. As for modeling, audiovisual material such as slides and pre-recorded videos could be used as examples to better orient the learner to the process. Such orientation and training may help stimulate the learner’s creation of story while allowing the training team to teach storytelling skills on a larger scale and with consistency. Additionally, training activities could include diverse media avenues to accommodate varying learning styles.^25^ The proportion of community members who attended the training was relatively small. Other training sessions should conduct targeted recruitment to ensure community representativeness. Finally, it would be important to assess the participants’ readiness to share and proceed with storytelling, and as the field is evolving, new tools are being developed to measure storytellers’ readiness. For example, questionnaires may serve as a self-reflective tool that prospective storytellers could use,^26^ and trainers can use quantitative ratings as a basis to gauge changes before and after the training workshop.

In conclusion, the ICTR’s storytelling training program was successfully implemented with consistently high satisfaction ratings and positive comments. The utility of storytelling as a culturally relevant experiential technique to potentially improve stakeholder engagement in the research process is to be determined. Future programs should address the impact of such training on actual engagement of participants’ storytelling abilities with stakeholders in the research process, from ideation of research questions to the dissemination of findings.
